# P-1367. Diagnostic Value of EBUS-TBNA in Sputum-Negative Tuberculosis: A Retrospective Five-Year Study

**DOI:** 10.1093/ofid/ofaf695.1554

**Published:** 2026-01-11

**Authors:** Ajithkumar Ittaman, Junais koleri, Moideenkutty Gurukkal, hamad Abdel Hadi, Muna Maslamani, A S H A ALEX, faraj Howady, reshna puthiyapura

**Affiliations:** HAMAD MEDICAL CORPORATION, Thrissur, Kerala, India; HAMAD MEDICAL CORPORATION, Thrissur, Kerala, India; HAMAD MEDICAL CORPORATION, Thrissur, Kerala, India; HAMAD MEDICAL CORPORATION, Thrissur, Kerala, India; HAMAD MEDICAL CORPORATION, Thrissur, Kerala, India; HAMAD MEDICAL CORPORATION, Thrissur, Kerala, India; HAMAD MEDICAL CORPORATION, Thrissur, Kerala, India; HAMAD MEDICAL CORPORATION, Thrissur, Kerala, India

## Abstract

**Background:**

Endobronchial ultrasound-guided transbronchial needle aspiration (EBUS-TBNA) has emerged as a valuable diagnostic tool for mediastinal lymphadenopathy, particularly in cases of sputum-negative tuberculosis (TB). This retrospective study evaluates the diagnostic accuracy of EBUS-TBNA in such cases, with subgroup analyses based on symptomatology and radiologic features

**Figure:**
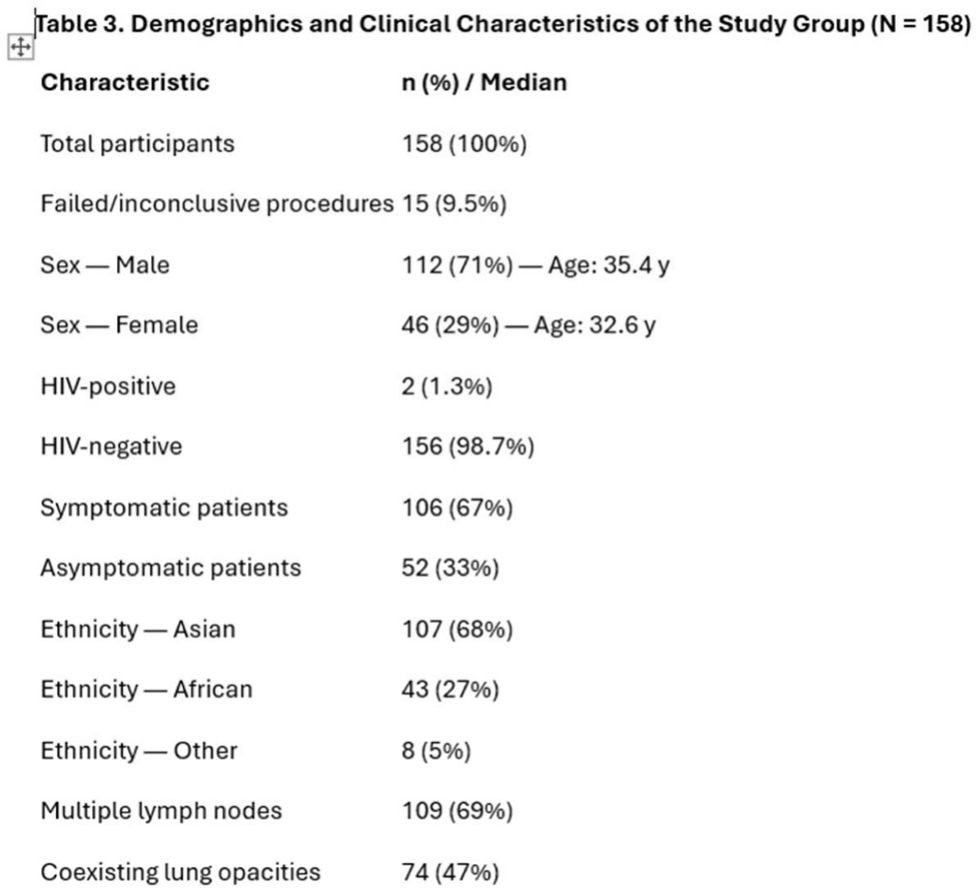
Demographics of study

**Figure:**
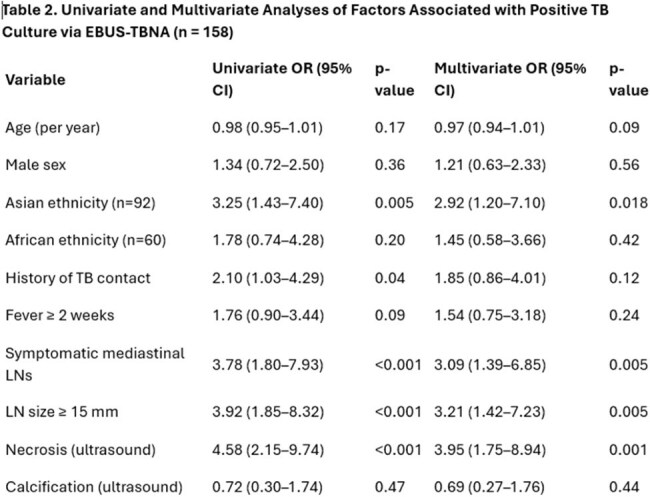
Analysis of sub groups

**Methods:**

A retrospective analysis was conducted on 158 patients with sputum-negative TB who underwent EBUS-TBNA between 2018 and 2023. Diagnostic metrics—including sensitivity, specificity, positive predictive value (PPV), and negative predictive value (NPV)—were calculated using microbiological culture results as the reference standard. Multivariate logistic regression was performed to identify predictors of positive TB culture. Subgroup analyses included symptomatic vs. asymptomatic mediastinal lymphadenopathy and presence/absence of coexisting lung lesions.

**Figure:**
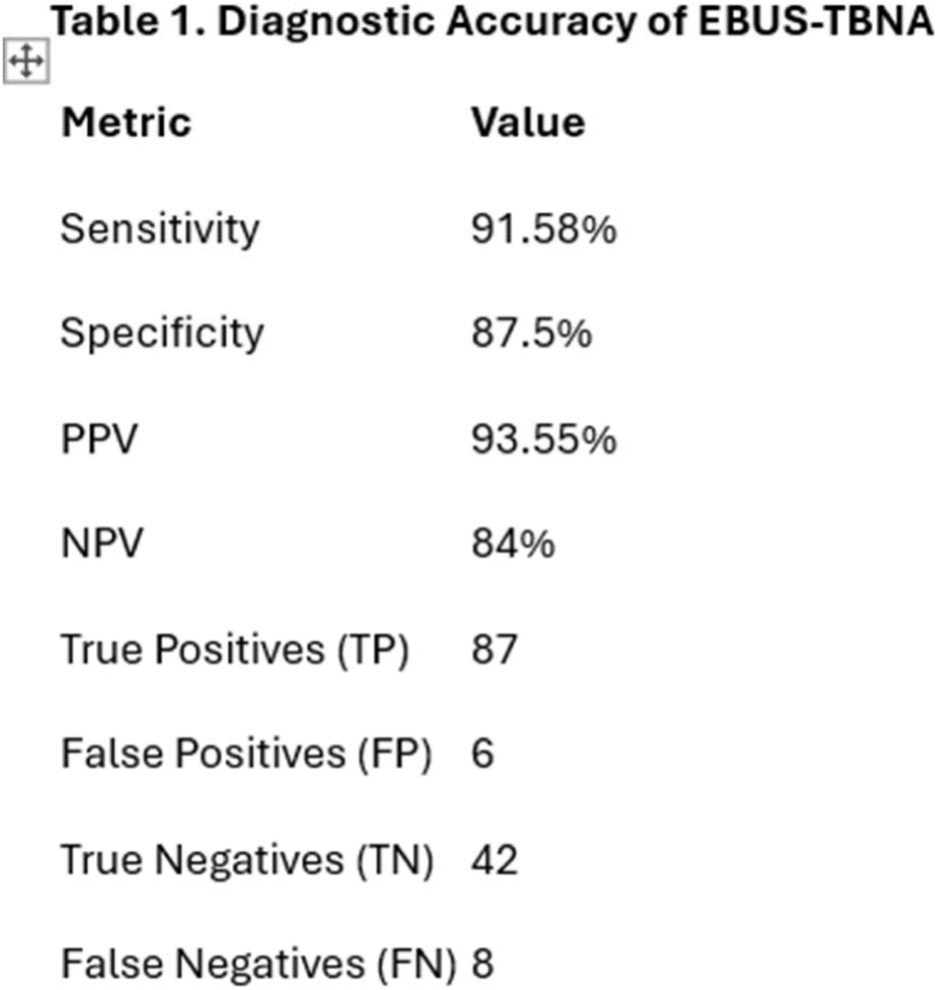
Result of study

**Results:**

In this five-year retrospective study highlight the high diagnostic accuracy of EBUS-TBNA in detecting sputum-negative tuberculosis. With a sensitivity of 91.58%, specificity of 87.5%, and a positive predictive value (PPV) of 93.55%, EBUS-TBNA reliably identified TB cases, achieving 87 true positives out of 158 patients. The negative predictive value (NPV) was also robust at 84%, with only 8 false negatives and 6 false positives. Multivariate analysis revealed that factors significantly associated with positive TB cultures included Asian ethnicity (OR: 2.92, *p*=0.018), symptomatic mediastinal lymphadenopathy (OR: 3.09, *p*=0.005), lymph node size ≥15 mm (OR: 3.21, *p*=0.005), and ultrasound-detected necrosis (OR: 3.95, *p*=0.001). Notably, 67% of patients were symptomatic, and nearly half exhibited coexisting lung lesions, underscoring the clinical complexity of sputum-negative TB.

**Conclusion:**

EBUS-TBNA demonstrates excellent diagnostic accuracy for sputum-negative TB, with a sensitivity of 91.58% and specificity of 87.5%. The procedure is particularly effective in symptomatic individuals and in those with necrotic or enlarged lymph nodes. These findings support the use of EBUS-TBNA as a frontline diagnostic modality in high-burden TB settings, especially when sputum studies are inconclusive

**Disclosures:**

All Authors: No reported disclosures

